# The basal epithelial marker P-cadherin associates with breast cancer cell populations harboring a glycolytic and acid-resistant phenotype

**DOI:** 10.1186/1471-2407-14-734

**Published:** 2014-10-01

**Authors:** Bárbara Sousa, Ana Sofia Ribeiro, Ana Rita Nobre, Nair Lopes, Diana Martins, Céline Pinheiro, André Filipe Vieira, André Albergaria, René Gerhard, Fernando Schmitt, Fátima Baltazar, Joana Paredes

**Affiliations:** IPATIMUP- Institute of Molecular Pathology and Immunology of the University of Porto, Rua Dr Roberto Frias s/n, Porto, 4200-465 Portugal; ICBAS- Institute of Biomedical Sciences Abel Salazar, University of Porto, Porto, Portugal; ICVS- Life and Health Sciences Research Institute, School of Health Sciences, University of Minho, Campus of Gualtar, Braga, Portugal; ICVS/3B’s - PT Government Associate Laboratory, Braga/Guimarães, Portugal; FMUP- Faculty of Medicine of the University of Porto, Porto, Portugal

**Keywords:** P-cadherin, Breast cancer, Hypoxia, Cancer stem cells

## Abstract

**Background:**

Cancer stem cells are hypoxia-resistant and present a preponderant glycolytic metabolism. These characteristics are also found in basal-like breast carcinomas (BLBC), which show increased expression of cancer stem cell markers.

Recently, we demonstrated that P-cadherin, a biomarker of BLBC and a poor prognostic factor in this disease, mediates stem-like properties and resistance to radiation therapy. Thus, the aim of the present study was to evaluate if P-cadherin expression was associated to breast cancer cell populations with an adapted phenotype to hypoxia.

**Methods:**

Immunohistochemistry was performed to address the expression of P-cadherin, hypoxic, glycolytic and acid-resistance biomarkers in primary human breast carcinomas. *In vitro* studies were performed using basal-like breast cancer cell lines. qRT-PCR, FACS analysis, western blotting and confocal microscopy were used to assess the expression of P-cadherin after HIF-1α stabilization, achieved by CoCl_2_ treatment*.* siRNA-mediated knockdown was used to silence the expression of several targets and qRT-PCR was employed to evaluate the effects of P-cadherin on HIF-1α signaling*.* P-cadherin high and low breast cancer cell populations were sorted by FACS and levels of GLUT1 and CAIX were assessed by FACS and western blotting. Mammosphere forming efficiency was used to determine the stem cell activity after specific siRNA-mediated knockdown, further confirmed by western blotting.

**Results:**

We demonstrated that P-cadherin overexpression was significantly associated with the expression of HIF-1α, GLUT1, CAIX, MCT1 and CD147 in human breast carcinomas. *In vitro*, we showed that HIF-1α stabilization was accompanied by increased membrane expression of P-cadherin and that P-cadherin silencing led to a decrease of the mRNA levels of *GLUT1* and *CAIX*. We also found that the cell fractions harboring high levels of P-cadherin were the same exhibiting more GLUT1 and CAIX expression. Finally, we showed that P-cadherin silencing significantly decreases the mammosphere forming efficiency in the same range as the silencing of HIF-1α, CAIX or GLUT1, validating that all these markers are being expressed by the same breast cancer stem cell population.

**Conclusions:**

Our results establish a link between aberrant P-cadherin expression and hypoxic, glycolytic and acid-resistant breast cancer cells, suggesting a possible role for this marker in cancer cell metabolism.

**Electronic supplementary material:**

The online version of this article (doi:10.1186/1471-2407-14-734) contains supplementary material, which is available to authorized users.

## Background

The tumor microenvironment is markedly defective on oxygen and nutrients, which seems to have a huge influence on the selection and survival of cancer stem cell populations. In fact, it is widely accepted that developing embryos, as well as regions of tissues with cells harboring stem cell properties (stem cell niches), usually present low oxygen tensions, suggesting hypoxia as a crucial event to maintain the undifferentiated state of stem/progenitor cells [1, 2]. Additionally, it is already widely accepted that undifferentiated cells, such as human embryonic stem cells (hESC) and induced-pluripotent stem cells (iPSC), present a glycolytic phenotype, decreased oxidative phosphorylation and ROS (reactive oxygen species) production, as well as altered lipid metabolism, when compared to their normal differentiated counterparts [3].

Cancer cells with stem-like properties, also known as cancer stem cells (CSC) or tumor-initiating cells (TICs), are also thought to reside in hypoxic niches within the tumor [2], exhibiting a metabolic program that allows their survival in this aggressive microenvironment [3]. This metabolic reprogramming is now recognized as a hallmark of cancer [4] and several players involved in cell metabolism are currently being considered as targets for cancer therapy [5].

HIF-1α, either induced by hypoxia or by alterations in oncogenes and/or tumor suppressor genes, induces the expression of gene products responsible for mediating changes in energy metabolism, pH regulation, angiogenesis, survival, invasion and motility [6]. In breast cancer, HIF-1α expression is associated with high-grade tumors, loss of estrogen receptor (ER), increased proliferation levels, decreased disease-free and overall patient survival and also with chemo- and radiotherapy resistance [7]. Moreover, cancer cells usually require increased glucose consumption, achieved by enhanced expression of glucose transporters (such as GLUT1). The increased glycolysis leads to intracellular acidosis that is controlled by upregulation of other membrane transporters, such as carbonic anhydrase IX (CAIX), monocarboxylate transporters (MCT1 and MCT4) and CD147/EMMPRIN (an extracellular matrix metalloproteinase inducer, required for proper location and function of MCT1 and MCT4).

Interestingly, the adaptation of cancer cells to limited oxygen availability, altered glucose metabolism and extracellular acidosis, are linked to a poor patient prognosis in breast cancer [8–10]. Chen *et al.* demonstrated that hypoxia, glycolytic and acid-resistant phenotypes are a powerful tumor cellular advantage and are associated to an aggressive behavior of breast carcinomas [11]. Moreover, several studies have been reporting that basal-like breast carcinomas (BLBC) show a stronger response to hypoxia [8, 10], as well as a higher glycolytic metabolism, than tumors with luminal characteristics [12–16]. In fact, triple-negative and HER2-overexpressing breast carcinomas present the higher tissue glucose metabolism, measured by 18 F-FDG PET scan, in comparison with the other breast cancer molecular subtypes [17], reinforcing the association between glycolytic metabolism and breast cancer poor prognosis.

P-cadherin, a calcium dependent cell-cell adhesion molecule encoded by the *CDH3* gene, is a protein whose expression is highly associated with undifferentiated cells in normal adult epithelial tissues, as well as with poorly differentiated carcinomas [18]. Its expression has been already reported in hESCs and is presumed to be a marker of stem or progenitor cells in epithelial tissues [19, 20]. During normal breast development, P-cadherin has a crucial role in the ductal mammary branching, being expressed by the monolayer of epithelial cap cells at the terminal end buds (TEBs) [21]. Moreover, this molecule is important for the undifferentiated state of the normal mammary gland [19], with its expression being restricted to the myoepithelium, although it has been postulated that it may be also present in early luminal progenitor cells [18, 22, 23]. In breast cancer, P-cadherin is aberrantly expressed in high-grade *in situ* [24] and invasive tumors [25], being a well-established indicator of poor patient prognosis [18, 22, 26]. The expression of P-cadherin is significantly associated with basal-like molecular subtype [25], which is mainly characterized by a triple-negative phenotype [ER, progesterone receptor (PgR) and HER2 negativity] and by the expression of basal/myoepithelial markers [27].

Moreover, P-cadherin expression promotes oncogenic-associated effects in breast cancer [18, 22] *.* Using *in vitro* and *in vivo* models, we demonstrated that it induces tumorigenesis, as well as cancer cell invasion partially through the secretion of matrix metalloproteinases (MMPs), such as MMP1 and MMP2 [28]. We have also disclosed that P-cadherin functional role is dependent on E-cadherin cellular context, since it interferes with the endogenous cadherin/catenin complex, inducing p120-catenin cytoplasmic delocalization and the consequent associated alterations in the actin cytoskeleton [29].

Recently, our group demonstrated that P-cadherin expression is associated with breast cancer stem cell markers, namely CD44, CD49f and ALDH1 [30]. We revealed that highly tumorigenic P-cadherin-enriched breast cancer cell populations harbor increased survival, resistance to radiation and stem cell properties [30]. Additionally, since it is accepted that breast cancer stem cells are pro-glycolytic [31] and more resistant to radiotherapy regimens [32], we hypothesized that the expression of P-cadherin could be associated to cell populations with an adapted phenotype to hypoxia and altered metabolism. Interestingly, by the analysis of an online available gene expression profile (E-GEOD-9649) [9], we could observe that *CDH3* gene is indeed upregulated in hypoxia compared to normoxic conditions, as well as in response to lactic acidosis.

In this work, we demonstrate that there is a significant association between aberrant P-cadherin expression and hypoxic, glycolytic and acid-resistant breast cancer cells, suggesting a role for this epithelial basal marker in cancer cell metabolism.

## Methods

### Breast tumor samples

Formalin-fixed, paraffin-embedded tissues of 473 invasive breast carcinomas in Tissue Microarrays (TMAs) were retrieved from the histopathology files of three Departments of Pathology: University Hospital of the Federal University of Santa Catarina (UFSC, Florianópolis, Brazil), Hospital Divino Espírito Santo (Ponta Delgada, Portugal) and from a private Laboratory of Pathology (Veronese Patologia e Citologia Araçatuba, Brazil). The tumors have been characterized for clinical and pathological features (data are summarized in Table S1 in Additional file 1). Molecular characterization of this series was previously studied and described [27]. This study was approved by the UFSC Ethics Committee for Human Research (CEPSH), by the Ethics Committee for Health from the Hospital do Divino Espírito Santo de Ponta Delgada E.P.E., as well as by the research review boards from the Veronese Patologia e Citologia Araçatuba Pathology Laboratory. Patients have signed a written informed consent, which implies that the spare biological material, which has not been used for diagnosis, can be used for research. This is in accordance with the national regulative law for the handling of biological specimens from tumor banks, being the samples exclusively available for research purposes in retrospective studies, as well as under the international Helsinki declaration.

### Cell culture

Human breast cancer cell lines were obtained as follows: BT20 was acquired from American Type Culture Collection (Manassas, VA, USA) and SUM149 was kindly provided by Dr. Stephen Ethier (University of Michigan, USA). Cells were routinely maintained at 37°C, 5% CO_2_, in the following media (Invitrogen Ltd, UK): DMEM for BT20 and 50% DMEM/50% Ham-F12 for SUM149. In BT20, the media contained 10% heat-inactivated fetal bovine serum, FBS, (Greiner bio-one, Belgium) and in SUM149 cell line, media was supplemented with 5% FBS, 5 μg/ml of insulin and 1 μg/ml of hydrocortisone (Sigma-Aldrich, USA). All media were supplemented with 100 IU/ml penicillin and 100 mg/ml streptomycin (Invitrogen Ltd, UK).

### Primary antibodies

The following primary anti-human antibodies were used for western blot (WB), immunohistochemistry (IHC) and flow cytometry (FC) against: P-cadherin [clone 56, BD Transduction Biosciences, USA; diluted 1:500 (WB) and 1:50 (IHC)], and APC-conjugated P-cadherin, R&D, USA; diluted 1:10 (FC)], HIF-1α [clone 54, BD Transduction Biosciences, USA; diluted 1:500 (WB) and 1:50 (IHC)], CAIX [ab15086, AbCam, Cambridge, UK; diluted 1:1000 (WB), 1:2000 (IHC) and 1:10 (FC)], Glut1 [ab15309, AbCam, Cambridge, UK; diluted 1:400 (WB), 1:500 (IHC) and 1:10 (FC)], MCT1 [AB3538P, Chemicon International, USA; diluted 1:200 (IHC)], MCT4 [AB3316P, Chemicon International, USA; diluted 1:100 (IHC)], CD147 [18-7344, Zymed Laboratories Inc., USA; diluted 1:750 (IHC)], GAPDH [0411, Santa Cruz Biotechnologies, USA; diluted 1:10000 (WB)] and β-actin [clone I-19, Santa Cruz Biotechnologies, USA; diluted 1:1000 (WB)].

### Immunohistochemistry

The immunohistochemical assays were performed with specific antibodies for P-cadherin, HIF-1α, GLUT1, CAIX, MCT1, MCT4 and CD147. Details about experimental procedures, primary antibodies, antigen retrieval detection systems and scoring are described elsewhere [12, 13, 27]. Specifically, HIF-1α immunohistochemistry was performed using CSA, Catalyzed Signal Amplification System (DAKOCytomation, USA), according to manufacturer’s instructions. Reactions were independently evaluated by two pathologists (FS and RG). All the proteins showed membrane staining, consistent with their cellular function, except for HIF-1α, which presented a nuclear pattern of expression and was considered positive whenever any strong and dark nuclear staining was observed. For the different antibodies studied, some samples could not be evaluated for the 473 cases of the series due to TMA’s cores missing or to insufficient representation of the tumor in the TMA core.

### siRNA transfection

Gene silencing was performed with validated siRNA, specific for *CDH3* (50nM, Hs_*CDH3*_6), HIF-1α (50nM, Hs_HIF1A_5), *GLUT1* (100nM Hs_*SLC2A1*_2) and *CAIX* (50nM, Hs_*CA9*_2). All siRNAs were from Qiagen (USA). Transfections were carried out using Lipofectamine 2000 (Invitrogen, UK), according to manufacturer’s recommended procedures. After incubation for 5 minutes, the siRNA and Lipofectamine 2000 solutions were mixed, incubated for additional 20 minutes and added to cell culture medium. A scrambled siRNA sequence, with no homology to any gene, was used as a negative control (Qiagen, USA). Gene inhibition was evaluated after 48 hours of cell transfection for *CDH3,* HIF-1α and *GLUT1* and after 72 hours for *CAIX*.

### CoCl_2_ treatment

Breast cancer cells were plated in T25 flasks for qRT-PCR and FACS, or in coverslips for immunofluorescence. After 24 hours, cells were treated with 200uM CoCl_2_ (Cobalt(II) chloride hexahydrate, Sigma-Aldrich, USA) diluted in ethanol for 4 hours.

### RNA extraction and qRT-PCR

RNA extraction was performed using RNeasy Mini Kit (Qiagen, USA) and cDNA was synthesized using the Omniscript Reverse Transcription kit (Qiagen, USA), following the manufacturer’s instructions. Quantitative-Real-Time-PCR (qRT-PCR) reaction was performed with TaqMan Gene Expression Assays (Applied Biosystems, USA), using gene-specific IDT probes (Integrated DNA Technologies, Inc., USA): *CDH3* (Hs.PT.51.5028751), *GLUT1* (Hs.PT.47.19044492.g), *CAIX* (Hs.PT.47.14063.g), and *GAPDH* (Hs.PT.39a.22214836). Analysis was performed with the ABI PRISM 7700 Sequence Detection System Instrument and software (Applied Biosystems, USA), following the manufacturer's recommendations. The internal standard human *GAPDH* was used to normalize cDNA quantity. Data was analyzed by the comparative 2(-ΔΔCT) method [33]. For all data comparisons, the Student's t-Test was used (two tailed, unequal variance). All reactions were done in triplicate and the results presented as mean of the values from three or more independent experiments.

### Immunofluorescence and confocal microscopic analysis

Cells were cultured on glass coverslips and 24 hours later they were treated either with 200uM of CoCl_2_ or with the respective vehicle (Ethanol) during 4 hours. After that, cells were fixed with 4% paraformaldehyde (20 minutes), treated with NH4Cl (50 mM) for 10 minutes, washed with PBS, and permeabilized with 0.1% Triton X-100 in PBS for 5 minutes, at room temperature. Unspecific reactions were blocked with 30 minutes incubation of cells with blocking solution (5% BSA in PBS-tween 0,5%). Cells were then stained with the primary antibodies, followed by incubation in the dark of Alexa488 or Alexa-594-conjugated secondary IgG (Dako Cytomation, Carpinteria, CA) in a 1:500 dilution. Primary and secondary antibodies were diluted in blocking solution. Each sample was mounted with Vectashield (Vector Laboratories, Inc, Burlingame, CA) containing DAPI and visualized with Leica SP5 confocal microscope (Leica Microsystems GmbH, Germany). Volume of cells of both conditions was acquired by Z-stack measurements.

### FACS analysis and sorting

For FACS analysis, cells were harvested with versene/0.48 mM EDTA (Invitrogen, UK), washed with PBS supplemented with 0.5% FBS and re-suspended in the stain buffer (2 mM EDTA and 0.5% bovine albumin in PBS). Single cell suspension was labeled with APC-conjugated P-cadherin, GLUT1 and CAIX antibodies. Cells transfected with the control siRNA and with *CDH3* siRNA were doubled stained either with P-cadherin and GLUT1 or CAIX antibodies. A live-dead stain (Invitrogen, UK) and the primary and secondary antibodies were incubated at 4°C, in the dark, for 15 minutes. Secondary Alexafluor-488-conjugated goat anti-rabbit IgG (Invitrogen, UK) was used in a 1:100 dilution. The labeled cells were then washed in the stain buffer and analyzed on a FACS Canto-II (BD Biosciences, USA).

For the sorting experiments, the subpopulations of SUM149 and BT20 breast cancer cells were selected according to P-cadherin expression (highest and lowest 20% expressing cells). Cells were sorted using BD Influx or FACS ARIA-II (BD Biosciences) and collected into 10% Hanks buffered solution (Invitrogen, UK). The purity of sorted populations was 80-95%. In addition, a further sample was also collected of cells passed through the laser under pressure, but not sorted, to act as a control for the effect of the pressure on the cells.

### Protein extraction and western blot analysis

Protein lysates were prepared from sorted cells, using catenin lysis buffer [1% (v/v) Triton X-100 and 1% (v/v) NP-40 (Sigma-Aldrich, USA) in deionized phosphate-buffered saline (PBS)] supplemented with 1:7 proteases inhibitors cocktail (Roche Diagnostics GmbH, Germany) for 10 min, at 4°C. Cell lysates were mixed with a vortex and centrifuged at 14000 rpm at 4°C, during 10 min. Supernatants were collected and protein concentration was determined using the Bradford assay (BioRad Protein Assay kit, USA). Proteins were dissolved in sample buffer [Laemmli with 5% (v/v) 2-b-mercaptoethanol and 5% (v/v) bromophenol blue] and boiled for 5 min at 95°C or at 65° (for GLUT1 staining). Samples were separated by SDS–PAGE and proteins were transferred into nitrocellulose membranes [Amersham Hybond enhanced chemiluminescence (ECL)]. For immunostaining, membranes were blocked with 5% (w/v) non-fat dry milk in PBS containing 0.5% (v/v) Tween20 and incubated during 1 hour with anti-P-cadherin and anti-GAPDH, two hours with anti- CAIX and anti- GLUT1 and overnight with anti-HIF-1α. After washes with PBS-Tween20, membranes were incubated with HRP-conjugated anti-mouse, goat or rabbit secondary antibodies (Santa Cruz Biotechnologies, USA) diluted 1:2000 for one hour. Proteins were then detected using ECL reagent (Amersham, USA) as a substrate. Quantity One software (BioRad, USA) was used for quantification of the differences in protein expression comparing with GAPDH expression.

### Mammosphere forming efficiency (MFE) assay

After the 72 h of the siRNA transfection, cells were enzymatically harvested and manually disaggregated with a 25-gauge needle to form a single-cell suspension and resuspended in cold PBS. Cells were plated at 500/cm2 in nonadherent culture conditions, in flasks coated with 1.2% poly(2-hydroxyethylmethacrylate)/95%ethanol (Sigma-Aldrich, USA) and allowed to grow for 5 days, in DMEM/F12 containing B27 supplement, and 500 ng/ml hydrochortisone, 40 ng/ml insulin, 20 ng/ml EGF in a humidified incubator at 37°C and 5% (v/v) CO2. Mammosphere forming efficiency was calculated as the number of mammospheres (≥50 μm) formed divided by the cell number plated, being expressed as a percentage.

### Statistical analysis

For immunohistochemistry results on breast cancer samples, statistical analysis was performed by SPSS statistics 17.0 software (SPSS Inc., USA). χ^2^ test and contingency tables were used to determine associations between groups and the results were considered statistically significant when the p-value was lower than 0.05. Concerning the functional *in vitro* assays, all were performed independently and in triplicate. Data were analyzed using the unpaired Student *t* test and considered statistically significant when the *P* value was less than 0.05. Statistical analyses were performed using Office Excel 2010 (Microsoft Corporation, Reading, UK). All statistical tests were two-sided.

## Results

### P-cadherin overexpression is significantly associated with the expression of hypoxic, glycolytic and acidosis markers in primary invasive breast carcinomas

**Figure 1:**
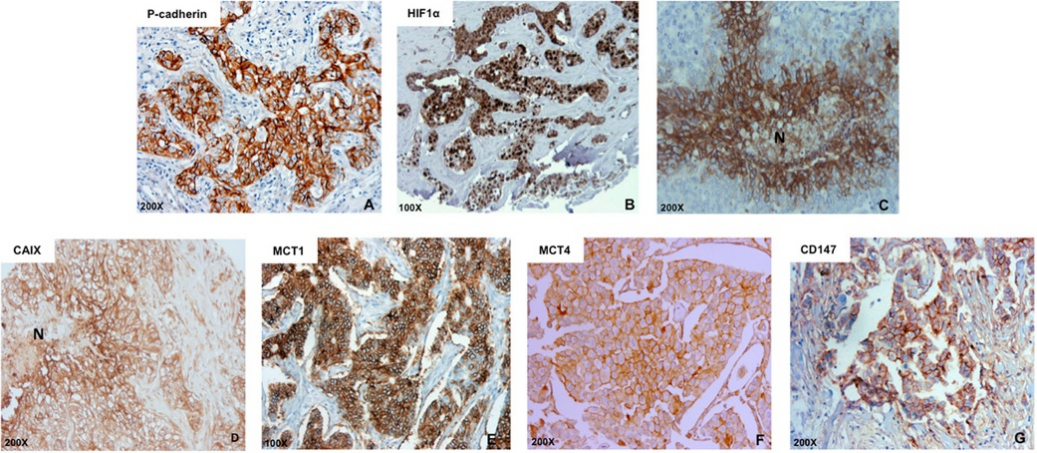
**Immunoexpression of P-cadherin, HIF-1α, GLUT1, CAIX, MCT1, MCT4 and CD147 in breast cancer samples.** Immunohistochemical staining for P-cadherin **(A)**, HIF-1α **(B)**, GLUT1 **(C)**, CAIX **(D)**, MCT1 **(E)**, MCT4 **(F)** and CD147 **(G)** expression in primary invasive breast carcinomas. Images **A**, **C**, **D**, **F** and **G** are in 200x magnification; **B** and **E** are in 100X magnification. N: necrosis.

In a large series of invasive breast carcinomas, previously classified for molecular subtypes [27], immunohistochemistry staining was performed for P-cadherin, HIF-1α, GLUT1, CAIX, MCT1, MCT4, and CD147 (Figure 1). Membrane P-cadherin expression was found in 145/468 (31%) of the cases. Nuclear HIF-1α was considered positive in 104/315 (33%) carcinomas. Concerning the membrane expression of GLUT1, CAIX, MCT1, MCT4 and CD147, we observed 140/327 (42.8%), 66/316 (20.8%), 106/407 (26%), 69/419 (16.5%) and 24/217 (11%) positive cases, respectively. Membrane GLUT1 and CAIX expression was frequently detected in peri-necrotic tumor areas (Figure 1).

The association between the expression of each one of these markers with the classical breast cancer prognostic factors (Table S2 in Additional file 1) as well as with the molecular subtypes and biomarkers ER, PgR, HER2 and Ki67 (Table S3 in Additional file 1) was evaluated. As previously reported, P-cadherin expression was significantly associated with high-grade carcinomas (*p < 0.0001*), HER2-overexpressing and basal-like molecular subtypes (*p < 0.0001*), ER and PgR negativity (*p < 0.0001*), high expression of HER2 (*p < 0.0001*), as well as with high Ki67 (*p = 0.0141*). Accordingly, HIF-1α expression was also associated with grade III (*p < 0.0001*) and high proliferative (*p = 0.0197*) tumors. Concerning the expression of GLUT1, CAIX, MCT1 and CD147, all have been significantly associated with high-grade (*p < 0.001*), basal-like (*p < 0.001*), ER and PgR negative (*p < 0.05*) tumors; absence of lymph node metastasis was more frequently observed in MCT1 expressing tumors (*p = 0.0223*) and CAIX expression was associated with an increased tumor size (*p = 0.0005*). Additionally, the expression of GLUT1, MCT1 and CD147 was associated with high proliferation indexes measured by Ki67 expression (*p = 0.0339*, *p = 0.0297*, *p = 0.0179*, respectively). There was still an expected significant association between the expression of hypoxic, glycolytic and acid-resistant phenotype markers (Table S4 in Additional file 1).

**Figure 2:**
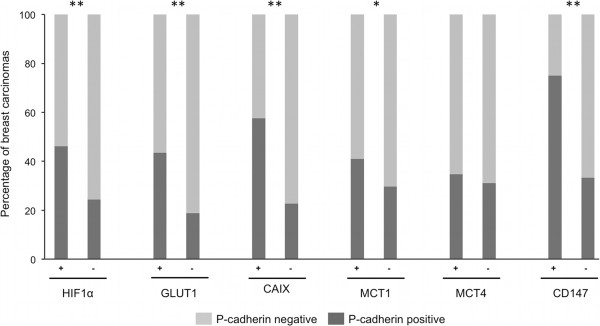
**Aberrant P-cadherin expression in HIF-1α, GLUT1, CAIX, MCT1, MCT4 and CD147 expressing breast carcinomas.** P-cadherin overexpression was significantly associated with the expression of HIF-1α, GLUT1, CAIX, MCT1 and CD147. No association was found with MCT4 expression. *p < 0.05; **p < 0.0001.

Interestingly, P-cadherin overexpression was also significantly associated with the expression of HIF-1α (*p < 0.0001*), GLUT1 (*p < 0.0001*), CAIX (*p < 0.0001*), MCT1 (*p = 0.0337*) and CD147 (*p < 0.0001*) (Table S5 in Additional file 1); in contrast, no association was found with MCT4 expression *(p = 0.553)*. With this data, we could demonstrate that breast carcinomas with positive expression to HIF-1α, GLUT1, CAIX, MCT1 and CD147 are significant associated to tumors showing a high percentage of cancer cells stained for the basal epithelial marker P-cadherin (Figure 2).

### HIF-1α stabilization by CoCl_2_ increases membrane P-cadherin expression in breast cancer cells

**Figure 3:**
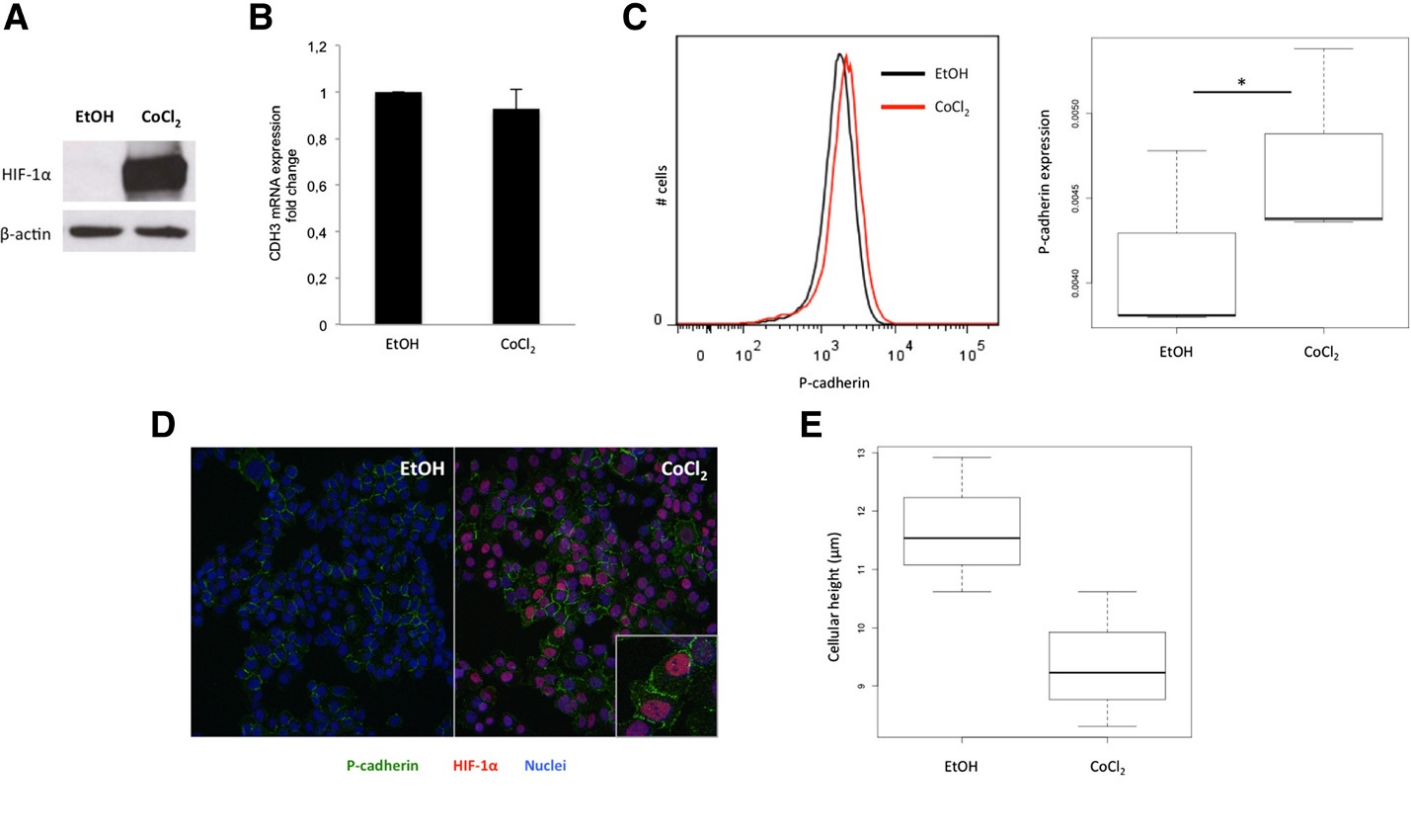
**HIF-1α stabilization induces the expression of P-cadherin in the membrane of SUM149 breast cancer cells.** HIF-1α stabilization and accumulation by CoCl_2_ treatment was confirmed by western blot **(A)**. qRT-PCR showed no significant alterations (*p = 0.562*) in *CDH3* mRNA levels upon CoCl_2_ treatment **(B)**. Using FACS analysis, a statistically significant increase (*p = 0.0246*) was observed in the expression of membrane P-cadherin in CoCl_2_ treated cells, when compared with the control cells treated with the vehicle (EtOH) **(C)**. Immunofluorescence of CoCl_2_ treated cells showed nuclear HIF-1α expression (red) and an increase of membrane P-cadherin expression (green), compared with vehicle treated cells **(D)**. Z-stack measurements revealed a decrease in the height of CoCl_2_-treated cells comparing with the control cells **(E)**. *p < 0.05.

Due to the direct association found between P-cadherin and HIF-1α expression in invasive breast carcinomas, as well as to the described association between both markers and the maintenance of stem-like properties, we decided to evaluate if HIF-1α stabilization could be affecting the expression of P-cadherin in breast cancer cells. Furthermore, by the use of a bioinformatics prediction tool [34, 35], we were able to recognize a putative binding site for HIF-1 transcription factor, positioned within a CpG island of *CDH3*/P-cadherin promoter [36]. Thus, we treated SUM149 breast cancer cells with CoCl_2_ in order to increase the expression of HIF-1α (Figure 3A). Although there were no alterations in *CDH3* mRNA levels (*p = 0.562*, Figure 3B), we could observe, by FACS analysis, a statistically significant increase of membrane P-cadherin expression upon HIF-1α stabilization (*p = 0.0246*; Figure 3C). We confirmed this result by confocal microscopy (Figure 3D), where we noticed that CoCl_2_ treatment resulted in nuclear accumulation of HIF-1α, as well as in an increased expression of P-cadherin at the cell membrane, when compared with cells treated only with the vehicle (ethanol). Although not statistically significant (*p = 0.0716*), we also observed a decrease in the cellular height after CoCl_2_ treatment (Figure 3E), indicating a re-organization of the cytoskeleton after HIF-1α stabilization, which can be associated with the induction of P-cadherin expression.

### P-cadherin expression interferes with *GLUT1*and *CAIX*mRNA levels in breast cancer cells

**Figure 4:**
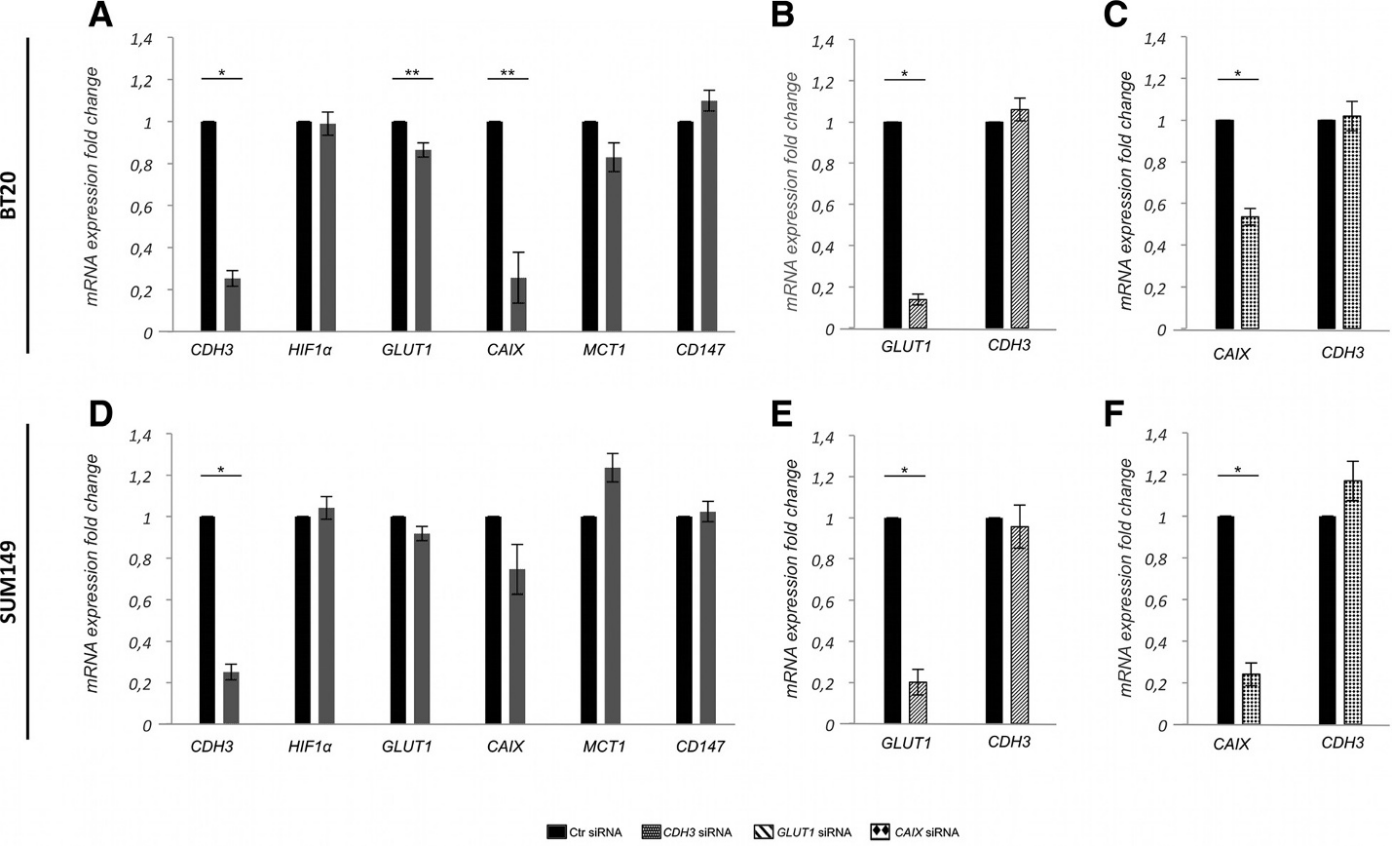
**P-cadherin expression affects** ***GLUT1*** **and** ***CAIX*** **mRNA levels in breast cancer cells.** mRNA expression measured by qRT-PCR of *CDH3*, *HIF-1α*, *GLUT1, CAIX, MCT1 and CD147* when inducing siRNA-mediated knockdown of *CDH3*  *, GLUT1*  and *CAIX*  in BT20 and SUM149 breast cancer cell lines. Upon *CDH3* silencing, there was a significant decrease of the mRNA expression of *GLUT1* and *CAIX* in BT20 **(A)** and a tendency, although not statistically significant, to a decrease in SUM149 cells **(D)**. No differences were observed in the mRNA expression of *HIF-1α, MCT1* and *CD147* in both cell lines. On the other hand, there were no alterations in *CDH3* mRNA expression in BT20 **(B and C)** and in SUM149 **(E and F)** breast cancer cell lines, when *GLUT1* **(B and E)** and *CAIX* **(C and F)** were silenced. *p < 0.05; **p < 0.0001.

The direct associations found in primary invasive breast carcinomas established, for the first time, a connection between P-cadherin expression and metabolic alterations of tumor cells. Therefore, in order to find out if these associations were reflecting a crosstalk between P-cadherin expression and the metabolic shift experienced by breast cancer cells, we decided to silence *CDH3* transcripts by siRNA-mediated knockdown in basal-like P-cadherin overexpressing breast cancer cell models (BT20 and SUM149). By real-time PCR, we could observe that *CDH3* silencing led to a statistically significant downregulation of *GLUT1* and *CAIX* mRNA in BT20 breast cancer cells (*p < 0.05*) (Figure 4A). Although not statistically significant, we could also find a tendency to a decrease in *GLUT1* and *CAIX* mRNA levels in SUM149 breast cancer cells (Figure 4D). No significant alterations were found in the mRNA expression of *HIF-1α, MCT1* and *CD147* upon *CDH3* silencing in both cell lines (Figure 4A and D). Interestingly, when *GLUT1* (Figure 4B and E) and *CAIX* (Figure 4C and F) were silenced, there were no significant alterations in *CDH3* mRNA levels.

### P-cadherin is co-expressed with GLUT1 and CAIX in basal-like breast cancer cell lines

**Figure 5:**
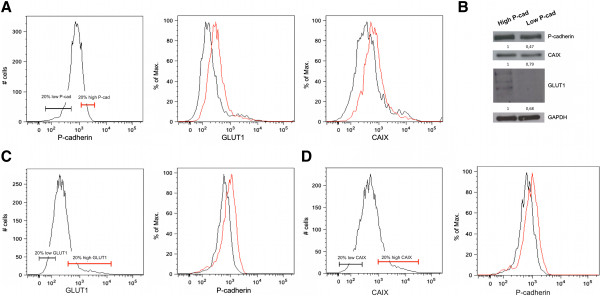
**P-cadherin is co-expressed with GLUT1 and CAIX in basal-like breast cancer cell lines.** By flow cytometry analysis, we observed that the 20% of cells with the highest and lowest P-cadherin expression presented highest and lowest, respectively, expression of GLUT1 and CAIX **(A)**. When cells were sorted by P-cadherin expression, lysed and analyzed in SDS-PAGE, the same result was observed concerning the high and low expression of GLUT1 and CAIX **(B)**. On the other hand, 20% of cells with the highest and lowest levels of GLUT1 and CAIX (**C** and **D**, respectively) expression also presented highest and lowest P-cadherin expression.

The above results led us to go further on the relationship between P-cadherin and GLUT1 and CAIX, since we observed that the expression of these both molecules was being somewhat responsive to P-cadherin in breast cancer cells. Thus, we decided to study if there was an enrichment of P-cadherin expression in GLUT1 and/or CAIX positive populations. Interestingly, we observed that cells presenting the highest expression of P-cadherin (20% high P-cad) were the ones also presenting the highest expression of GLUT1 and CAIX, while the ones showing the lowest expression of P-cadherin (20% low P-cad) demonstrated the lowest levels of GLUT1 and CAIX (Figure 5A). This result was confirmed when we sorted and separated the 20% high and low P-cadherin cell populations and analyzed the expression of GLUT1 and CAIX by western blot (Figure 5B). Furthermore, when we selected the population of cells by their GLUT1 expression, the 20% high/low GLUT1 cells also presented the highest and lowest levels of P-cadherin expression, respectively (Figure 5C). Still, the cells selected by CAIX expression also presented the same tendency concerning P-cadherin expression (Figure 5D). Similar results were obtained in BT20 breast cancer cells (Figure S1 in Additional file 2).

### The inhibition of *CDH3*, *HIF-1α*, *GLUT1*and *CAIX*affects MFE in basal-like breast cancer cells

**Figure 6:**
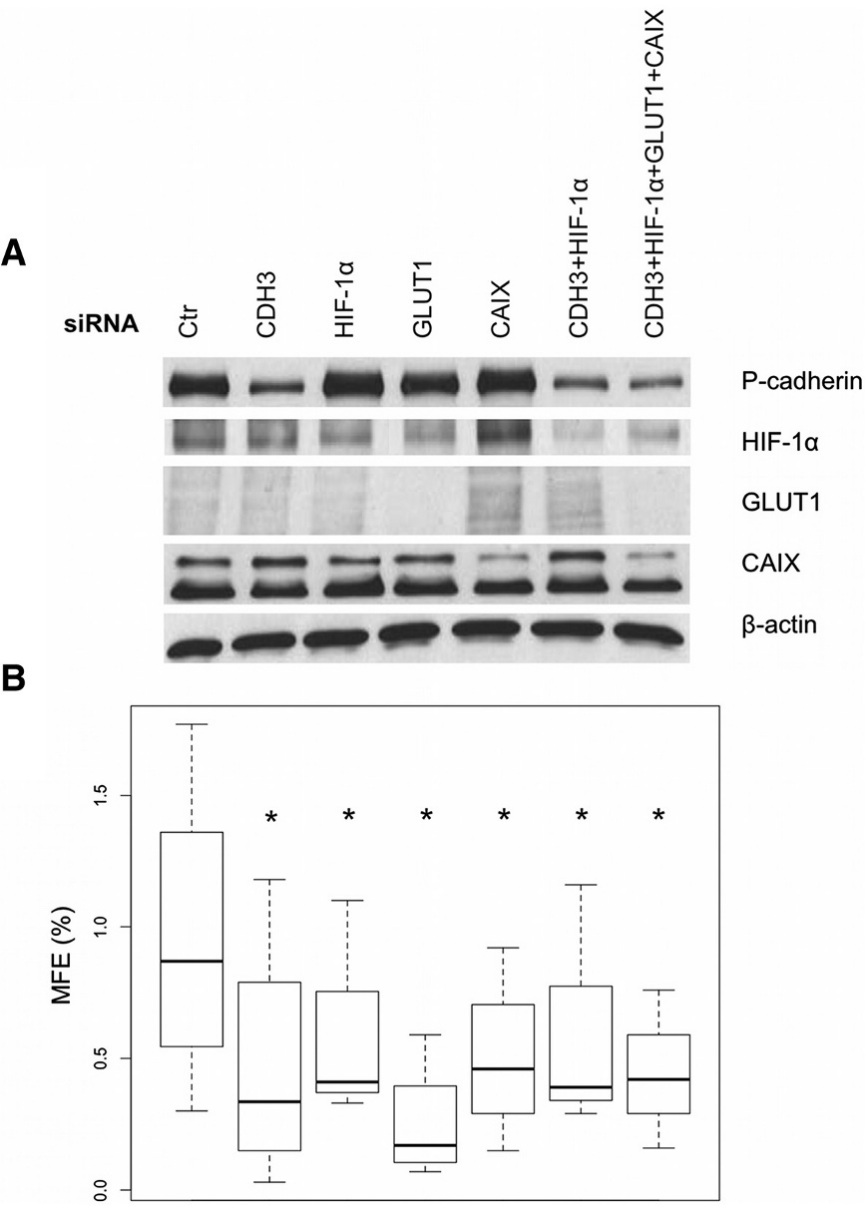
**MFE (%) decreases upon siRNA-mediated silencing of** ***CDH3*** **,** ***HIF-1α, GLUT1*** **and** ***CAIX*** **in SUM149 cells.** siRNA-mediated silencing of *CDH3, HIF-1α, GLUT1* and *CAIX* was confirmed by western blot analysis **(A)**. A statistically significant decrease in MFE (%) was observed when we silenced *CDH3, HIF-1α, GLUT1* and *CAIX.* The simultaneous silencing of *CDH3* and *HIF-1α* also revealed a significant decrease of MFE (%) of the target cells, although not cumulative. Still, the silencing of the expression of all transcripts tested led to a non-cumulative inhibition of the MFE compared to the control cells **(B)**. *p < 0.05.

Our previous results show that HIF-1α stabilization was accompanied by an increase of membrane P-cadherin expression, which was co-expressed with GLUT1 and CAIX in breast cancer cells. Since it has been already described that HIF-1α, GLUT1 and CAIX are required for CSC survival and tumor aggressiveness, and that we have recently shown that P-cadherin is also involved in the maintenance of stem-like properties of basal-like breast CSCs, we decided to evaluate the effect of the inhibition of all these molecules, alone or in combination, on the mammosphere forming efficiency (MFE%) of the SUM149 breast cancer cell model (Figure 6). As expected, silencing the expression of *CDH3*, as well as of *HIF-1α, GLUT1* and *CAIX*, showed a significant decrease of the ability to form mammospheres when compared with the cells transfected with the control siRNA (*p = 0.0153*, *p = 0.0156*, *p = 0.000284* and *p = 0.000902*, respectively). Moreover, when we simultaneously silenced *CDH3* and *HIF-1α*, we also observed a decrease of MFE of the target cells (*p = 0.0367*), although not cumulative. If, in addition to *CDH3* and *HIF-1α*, we silence the downstream targets *GLUT1* and *CAIX* (siRNA *CDH3 + HIF-1α + GLUT1 + CAIX*), there is still a non-cumulative decrease effect in MFE (%) (*p = 0.0152*).

## Discussion

Much attention has been paid to the tissue microenvironment, highlighting the importance of hypoxia as a mediator of cell survival, pluripotency, stemness and proliferation of normal stem cells [2, 3] Alterations in metabolism have also been proven to be important for the maintenance of stem cell properties [3]. hESC and iPSC tends be more glycolytic and less oxidative comparing to their somatic counterparts [37–39]. Interestingly, the behavior of some tumor cells resembles in many aspects the behavior of hESC, as well as adult stem cells [3].

P-cadherin has been recently described as a mediator of stem-like properties in basal-like breast cancer cell lines. The main goal of this work was to understand if there was an association between P-cadherin overexpression and the phenotype of adaptation of breast cancer cells to microenvironment stresses, such as hypoxia, lactic acidosis and glycolytic metabolism, also characteristics of the undifferentiated state of stem, progenitor and breast CSC. Interestingly, we showed that P-cadherin is associated to the hypoxic phenotype and metabolic reprogramming of breast cancer cells.

We demonstrated an association between the expression of P-cadherin and HIF-1α in breast carcinomas. Similarly to P-cadherin, HIF-1α expression is associated to worse prognosis in breast cancer, short patient’s survival, high proliferation and poor tumor differentiation [7, 40]. Interestingly, we found that HIF-1α stabilization promotes membrane P-cadherin expression in breast cancer cells, although no alterations were detected in *CDH3* mRNA levels after CoCl_2_ treatment. With these results, we can hypothesize that increased P-cadherin membrane expression observed after HIF-1α accumulation occurs at a post-transcriptional level, and not as a direct effect. Thus, based on our findings and on previous data reported by others, the crosstalk between these molecules might be explained by several hypotheses. Since hypoxia, through HIF-1α [41], is able to induce ER degradation and that ER is a transcription *CDH3* repressor [26, 42], it is possible that hypoxia can be inducing HIF-1α, promoting ER degradation and allowing *CDH3* transcription. Other possible explanation is related to the link between hypoxia and BRCA1, another *CDH3* transcriptional repressor [43]. It is known that hypoxia induces BRCA1 downregulation [44] and that *BRCA1*-mutated breast carcinomas, which are enriched in HIF-1α expression [45], present aberrant expression of P-cadherin [46].

Concomitantly with the increase of membrane P-cadherin expression, we still observed a decrease in the cellular height after CoCl_2_ stabilization, indicating a putative re-organization of the cytoskeleton and cell morphology, with the acquisition of a phenotype associated to breast cancer cell aggressiveness [47]. This observation is consistent with our previous data, showing that P-cadherin expression is able to induce invasion, migration and motility in breast cancer cells [28] and that its modulation also interferes with GTPase-mediated signal transduction and actin cytoskeleton organization [29].

Our results also showed an association between P-cadherin and GLUT1, CAIX, MCT1 and CD147. These molecules are important response mediators of cancer cell survival in stressful microenvironments and are upregulated in poor prognosis BLBC [12, 13], where P-cadherin is aberrantly expressed. Upregulation of membrane transporters, such as CAIX and MCTs, is responsible for the extracellular acidification, assisting *in vitro* cancer cell invasion and *in vivo* metastization through the acidic degradation of the ECM [48]. CD147/EMMPRIN is known to induce MMP production [49], such as MMP2 [50], to promote tumor growth, inhibit cell apoptosis and enhance cell migration under hypoxic conditions [51]. Interestingly, the highly significant association found between P-cadherin and CD147 is in accordance with the above-described reports and also with our previous data, showing that P-cadherin induces an invasive behavior in breast cancer cells through the activation of MMP1 and MMP2 [28].

Using basal-like breast cancer cell lines, we found that P-cadherin silencing is able to induce the downregulation of *GLUT1* and *CAIX* mRNA, whereas *GLUT1* and *CAIX* knockdown showed little or no effect in *CDH3* mRNA expression. Thus, although the expression variations found were not so prominent as expected for direct molecular targets, we believe that P-cadherin is putatively involved in a signaling pathway that interferes with the metabolic reprograming of cancer cells. Moreover, we also demonstrated that the cell subpopulation expressing more/less P-cadherin at the cell surface was the same presenting higher/lower levels of GLUT1 and CAIX and vice-versa. These results suggests that P-cadherin overexpressing breast cancer cells are most likely to exhibit increased glycolysis and to survive to metabolic-driven pH alterations, justifying the enhanced aggressive behavior and metastatic properties.

In breast cancer, hypoxia and HIF-1α have a pivot role in promoting tumor growth and metastasis through the maintenance, expansion as well as increased activity of breast CSCs [52–54]. Oliveira-Costa *et al.* showed that HIF-1α was differently expressed in CD44^+^/CD24^-/low^ breast cancer cells [55]. Moreover, CAIX also plays a role in the regulation of stemness and expansion of breast CSCs in hypoxic niches [56]. Recently, our group demonstrated that P-cadherin expression is able to mediate stem cell properties in basal-like breast cancer cells [30]. Still, we showed that BLBC, which are enriched in P-cadherin expression, present a high percentage of cells with CSC phenotype [57]. Accordingly, we demonstrated that the inhibition of *CDH3, HIF-1α, GLUT1* and *CAIX* in basal-like breast cancer cells significantly reduces their MFE, an important measure of breast cancer stem cell activity. We still show that the silencing of *CDH3* and *HIF-1α,* or even the simultaneous inhibition of *CDH3, HIF-1α, GLUT1* and *CAIX,* have a non-cumulative effect in the inhibition in MFE comparing to the single silencing of any of these molecules. This result is in accordance with our observation that the cell subpopulation expressing increased levels of P-cadherin also presents higher levels of GLUT1 and CAIX, demonstrating that we are targeting the same breast cancer stem cell population.

Taking together, we believe that the hypoxic environment in breast tumors might be selecting a pool of cancer cells with stem-like properties, showing increased P-cadherin expression as well as a distinct metabolic state. These characteristics will be accounted for therapeutic resistance, since breast CSCs are resistant to radiotherapy and are thought to be responsible for breast cancer metastasis [32, 58]. Interestingly, previous data from our group also demonstrated that P-cadherin inhibition sensitizes breast cancer cells to radiation-induced cell death [30].

## Conclusions

In conclusion, we demonstrated that aberrant P-cadherin expression is associated with the hypoxic/glycolytic and acid-resistant phenotype in invasive breast carcinomas, represented by a panel of markers including HIF-1α, GLUT1, CAIX, MCT1 and CD147. We also showed that membrane P-cadherin expression can be increased by HIF-1α stabilization, as well as can modulate *GLUT1* and *CAIX* expression. Moreover, we still demonstrated that P-cadherin is differentially expressed in basal-like breast cancer cells that also present higher levels of GLUT1 and CAIX. Thus, we believe that HIF-1α might be stabilizing membrane P-cadherin expression in breast CSC or even selecting P-cadherin-high expressing breast CSCs in the hypoxic stem cell niche. In its turn, P-cadherin expression is probably shifting the metabolic program of these cells, which might be responsible for tumor aggressiveness, as well as for their ability to survive, compared to the low P-cadherin expressing cells. In this case, P-cadherin is mediating the survival of aggressive cells thought to be resistant to standard cancer therapies, being responsible for tumor relapses and metastasis in breast cancer patients.

## Electronic supplementary material

Additional file 1: Table S1: Clinical, pathological and immunohistochemical characteristics of the 473 primary invasive breast carcinomas*. **Table S2.** Association of P-cadherin, HIF-1α, GLUT1, CAIX, MCT1, MCT4 and CD147 and classic prognostic factors in breast cancer*. **Table S3.** Association of biomarkers and classical molecular markers used in breast cancer*. **Table S4.** Association between the hypoxia, glycolytic and acid-resistant phenotype markers within the series of invasive breast carcinomas*. **Table S5.** P-cadherin overexpression is associated with the expression of markers of hypoxia, glycolytic and acid-resistant phenotype in breast cancer*. (DOCX 80 KB)

Additional file 2: Figure S1: P-cadherin is co-expressed with GLUT1 and CAIX in BT20 cell line. (PDF 179 KB)

## Abbreviations

BLBC: Basal-like breast carcinomas
CAIX: Carbonic anhydrase IX
CSC: Cancer stem cells
EMMPRIN: Extracellular matrix metalloproteinase inducer
ER: Estrogen receptor
GLUT1: Glucose transporter 1
hESC: Human embryonic stem cells
iPSC: Induced-pluripotent stem cells
MCT: Monocarboxylate transporter
MFE: Mammosphere forming efficiency
MMPs: Metalloproteinases
PgR: Progesterone receptor
ROS: Reactive oxygen species
TEBs: Terminal end buds
TICs: Tumor-initiating cells
TMA: Tissue microarrays.

## References

[1] Yochim JM, Mitchell JA (1968). Intrauterine Oxygen Tension in the Rat During Progestation: Its Possible Relation to Carbohydrate Metabolism and the Regulation of Nidation. Endocrinology.

[2] Mohyeldin A, Garzon-Muvdi T, Quinones-Hinojosa A (2010). Oxygen in stem cell biology: a critical component of the stem cell niche. Cell stem cell.

[3] Vacanti NM, Metallo CM (1830). Exploring metabolic pathways that contribute to the stem cell phenotype. Biochim Biophys Acta.

[4] Ward PS, Thompson CB (2012). Metabolic reprogramming: a cancer hallmark even warburg did not anticipate. Cancer Cell.

[5] Birsoy K, Wang T, Possemato R, Yilmaz OH, Koch CE, Chen WW, Hutchins AW, Gultekin Y, Peterson TR, Carette JE, Brummelkamp TR, Clish CB, Sabatini DM (2013). MCT1-mediated transport of a toxic molecule is an effective strategy for targeting glycolytic tumors. Nat Genet.

[6] Semenza GL (2003). Targeting HIF-1 for cancer therapy. Nat Rev Cancer.

[7] Lundgren K, Holm C, Landberg G (2007). Hypoxia and breast cancer: prognostic and therapeutic implications. Cell Mol Life Sci.

[8] Chi JT, Wang Z, Nuyten DS, Rodriguez EH, Schaner ME, Salim A, Wang Y, Kristensen GB, Helland A, Borresen-Dale AL, Giaccia A, Longaker MT, Hastie T, Yang GP, van de Vijver MJ, Brown PO (2006). Gene expression programs in response to hypoxia: cell type specificity and prognostic significance in human cancers. PLoS Med.

[9] Chen JL, Lucas JE, Schroeder T, Mori S, Wu J, Nevins J, Dewhirst M, West M, Chi JT (2008). The genomic analysis of lactic acidosis and acidosis response in human cancers. PLoS Genet.

[10] Gatza ML, Kung HN, Blackwell KL, Dewhirst MW, Marks JR, Chi JT (2011). Analysis of tumor environmental response and oncogenic pathway activation identifies distinct basal and luminal features in HER2-related breast tumor subtypes. Breast Cancer Res.

[11] Chen CL, Chu JS, Su WC, Huang SC, Lee WY (2010). Hypoxia and metabolic phenotypes during breast carcinogenesis: expression of HIF-1alpha, GLUT1, and CAIX. Virchows Arch.

[12] Pinheiro C, Albergaria A, Paredes J, Sousa B, Dufloth R, Vieira D, Schmitt F, Baltazar F (2010). Monocarboxylate transporter 1 is up-regulated in basal-like breast carcinoma. Histopathology.

[13] Pinheiro C, Sousa B, Albergaria A, Paredes J, Dufloth R, Vieira D, Schmitt F, Baltazar F (2011). GLUT1 and CAIX expression profiles in breast cancer correlate with adverse prognostic factors and MCT1 overexpression. Histol Histopathol.

[14] Tan EY, Yan M, Campo L, Han C, Takano E, Turley H, Candiloro I, Pezzella F, Gatter KC, Millar EK, O'Toole SA, McNeil CM, Crea P, Segara D, Sutherland RL, Harris AL, Fox SB (2009). The key hypoxia regulated gene CAIX is upregulated in basal-like breast tumours and is associated with resistance to chemotherapy. Br J Cancer.

[15] Dong C, Yuan T, Wu Y, Wang Y, Fan Teresa WM, Miriyala S, Lin Y, Yao J, Shi J, Kang T, Lorkiewicz P, St Clair D, Hung MC, Evers BM, Zhou BP (2013). Loss of FBP1 by Snail-Mediated Repression Provides Metabolic Advantages in Basal-like Breast Cancer. Cancer Cell.

[16] McCleland ML, Adler AS, Shang Y, Hunsaker T, Truong T, Peterson D, Torres E, Li L, Haley B, Stephan JP, Belvin M, Hatzivassiliou G, Blackwood EM, Corson L, Evangelista M, Zha J, Firestein R (2012). An integrated genomic screen identifies LDHB as an essential gene for triple-negative breast cancer. Cancer Res.

[17] Koo HR, Park JS, Kang KW, Cho N, Chang JM, Bae MS, Kim WH, Lee SH, Kim MY, Kim JY, Seo M, Moon WK (2014). 18 F-FDG uptake in breast cancer correlates with immunohistochemically defined subtypes. Eur Radiol.

[18] Albergaria A, Ribeiro AS, Vieira AF, Sousa B, Nobre AR, Seruca R, Schmitt F, Paredes J (2011). P-cadherin role in normal breast development and cancer. Int J Dev Biol.

[19] Radice GL, Ferreira-Cornwell MC, Robinson SD, Rayburn H, Chodosh LA, Takeichi M, Hynes RO (1997). Precocious mammary gland development in P-cadherin-deficient mice. J Cell Biol.

[20] Kolle G, Ho M, Zhou Q, Chy HS, Krishnan K, Cloonan N, Bertoncello I, Laslett AL, Grimmond SM (2009). Identification of human embryonic stem cell surface markers by combined membrane-polysome translation state array analysis and immunotranscriptional profiling. Stem Cells.

[21] Daniel CW, Strickland P, Friedmann Y (1995). Expression and functional role of E- and P-cadherins in mouse mammary ductal morphogenesis and growth. Develop Biol.

[22] Paredes J, Correia AL, Ribeiro AS, Albergaria A, Milanezi F, Schmitt FC (2007). P-cadherin expression in breast cancer: a review. Breast Cancer Res.

[23] Palacios J, Benito N, Pizarro A, Suarez A, Espada J, Cano A, Gamallo C (1995). Anomalous expression of P-cadherin in breast carcinoma. Correlation with E-cadherin expression and pathological features. Am J Pathol.

[24] Paredes J, Lopes N, Milanezi F, Schmitt FC (2007). P-cadherin and cytokeratin 5: useful adjunct markers to distinguish basal-like ductal carcinomas in situ. Virchows Arch.

[25] Paredes J, Albergaria A, Oliveira JT, Jeronimo C, Milanezi F, Schmitt FC (2005). P-cadherin overexpression is an indicator of clinical outcome in invasive breast carcinomas and is associated with CDH3 promoter hypomethylation. Clin Cancer Res.

[26] Paredes J, Milanezi F, Reis-Filho JS, Leitao D, Athanazio D, Schmitt F (2002). Aberrant P-cadherin expression: is it associated with estrogen-independent growth in breast cancer?. Pathol Res Pract.

[27] Sousa B, Paredes J, Milanezi F, Lopes N, Martins D, Dufloth R, Vieira D, Albergaria A, Veronese L, Carneiro V, Carvalho S, Costa JL, Zeferino L, Schmitt F (2010). P-cadherin, vimentin and CK14 for identification of basal-like phenotype in breast carcinomas: an immunohistochemical study. Histol Histopathol.

[28] Ribeiro AS, Albergaria A, Sousa B, Correia AL, Bracke M, Seruca R, Schmitt FC, Paredes J (2010). Extracellular cleavage and shedding of P-cadherin: a mechanism underlying the invasive behaviour of breast cancer cells. Oncogene.

[29] Ribeiro AS, Sousa B, Carreto L, Mendes N, Nobre AR, Ricardo S, Albergaria A, Cameselle-Teijeiro JF, Gerhard R, Soderberg O, Seruca R, Santos MA, Schmitt F, Paredes J (2013). P-cadherin functional role is dependent on E-cadherin cellular context: a proof of concept using the breast cancer model. J Pathol.

[30] Vieira AF, Ricardo S, Ablett MP, Dionisio MR, Mendes N, Albergaria A, Farnie G, Gerhard R, Cameselle-Teijeiro JF, Seruca R, Schmitt F, Clarke RB, Paredes J (2012). P-cadherin is coexpressed with CD44 and CD49f and mediates stem cell properties in basal-like breast cancer. Stem Cells.

[31] Feng W, Gentles A, Nair RV, Huang M, Lin Y, Lee CY, Cai S, Scheeren FA, Kuo AH, Diehn M (2014). Targeting unique metabolic properties of breast tumor initiating cells. Stem Cells.

[32] Phillips TM, McBride WH, Pajonk F (2006). The response of CD24(-/low)/CD44+ breast cancer-initiating cells to radiation. J Natl Cancer Inst.

[33] Livak KJ, Schmittgen TD (2001). Analysis of relative gene expression data using real-time quantitative PCR and the 2(-Delta Delta C(T)) Method. Methods.

[34] Messeguer X, Escudero R, Farre D, Nunez O, Martinez J, Alba MM (2002). PROMO: detection of known transcription regulatory elements using species-tailored searches. Bioinformatics.

[35] Farre D, Roset R, Huerta M, Adsuara JE, Rosello L, Alba MM, Messeguer X (2003). Identification of patterns in biological sequences at the ALGGEN server: PROMO and MALGEN. Nucleic Acids Res.

[36] Flicek P, Amode MR, Barrell D, Beal K, Billis K, Brent S, Carvalho-Silva D, Clapham P, Coates G, Fitzgerald S, Gil L, Giron CG, Gordon L, Hourlier T, Hunt S, Johnson N, Juettemann T, Kahari AK, Keenan S, Kulesha E, Martin FJ, Maurel T, McLaren WM, Murphy DN, Nag R, Overduin B, Pignatelli M, Pritchard B, Pritchard E, Riat HS (2014). Ensembl 2014. Nucleic Acids Res.

[37] Varum S, Rodrigues AS, Moura MB, Momcilovic O, Easley CA, Ramalho-Santos J, Van Houten B, Schatten G (2011). Energy metabolism in human pluripotent stem cells and their differentiated counterparts. PLoS One.

[38] Panopoulos AD, Yanes O, Ruiz S, Kida YS, Diep D, Tautenhahn R, Herrerias A, Batchelder EM, Plongthongkum N, Lutz M, Berggren WT, Zhang K, Evans RM, Siuzdak G, Izpisua, Belmonte JC (2012). The metabolome of induced pluripotent stem cells reveals metabolic changes occurring in somatic cell reprogramming. Cell Res.

[39] Folmes CD, Nelson TJ, Martinez-Fernandez A, Arrell DK, Lindor JZ, Dzeja PP, Ikeda Y, Perez-Terzic C, Terzic A (2011). Somatic oxidative bioenergetics transitions into pluripotency-dependent glycolysis to facilitate nuclear reprogramming. Cell Metab.

[40] Generali D, Berruti A, Brizzi MP, Campo L, Bonardi S, Wigfield S, Bersiga A, Allevi G, Milani M, Aguggini S, Gandolfi V, Dogliotti L, Bottini A, Harris AL, Fox SB (2006). Hypoxia-inducible factor-1alpha expression predicts a poor response to primary chemoendocrine therapy and disease-free survival in primary human breast cancer. Clin Cancer Res.

[41] Cooper C, Liu GY, Niu YL, Santos S, Murphy LC, Watson PH (2004). Intermittent hypoxia induces proteasome-dependent down-regulation of estrogen receptor alpha in human breast carcinoma. Clin Cancer Res.

[42] Paredes J, Stove C, Stove V, Milanezi F, Van Marck V, Derycke L, Mareel M, Bracke M, Schmitt F (2004). P-cadherin is up-regulated by the antiestrogen ICI 182,780 and promotes invasion of human breast cancer cells. Cancer Res.

[43] Gorski J, James C, Quinn J, Stewart G, Staunton K, Buckley N, McDyer F, Kennedy R, Wilson R, Mullan P, Harkin D (2010). BRCA1 transcriptionally regulates genes associated with the basal-like phenotype in breast cancer. Breast Cancer Res Treat.

[44] Lu Y, Chu A, Turker MS, Glazer PM (2011). Hypoxia-induced epigenetic regulation and silencing of the BRCA1 promoter. Mol Cell Biol.

[45] Yan M, Rayoo M, Takano EA, Thorne H, Fox SB (2009). BRCA1 tumours correlate with a HIF-1[alpha] phenotype and have a poor prognosis through modulation of hydroxylase enzyme profile expression. Br J Cancer.

[46] Arnes JB, Brunet JS, Stefansson I, Begin LR, Wong N, Chappuis PO, Akslen LA, Foulkes WD (2005). Placental cadherin and the basal epithelial phenotype of BRCA1-related breast cancer. Clin Cancer Res.

[47] Gest C, Joimel U, Huang L, Pritchard LL, Petit A, Dulong C, Buquet C, Hu CQ, Mirshahi P, Laurent M, Fauvel-Lafeve F, Cazin L, Vannier JP, Lu H, Soria J, Li H, Varin R, Soria C (2013). Rac3 induces a molecular pathway triggering breast cancer cell aggressiveness: differences in MDA-MB-231 and MCF-7 breast cancer cell lines. BMC Cancer.

[48] Swietach P, Vaughan-Jones RD, Harris AL (2007). Regulation of tumor pH and the role of carbonic anhydrase 9. Cancer Metastasis Rev.

[49] Gabison EE, Hoang-Xuan T, Mauviel A, Menashi S (2005). EMMPRIN/CD147, an MMP modulator in cancer, development and tissue repair. Biochimie.

[50] Zucker S, Hymowitz M, Rollo EE, Mann R, Conner CE, Cao J, Foda HD, Tompkins DC, Toole BP (2001). Tumorigenic potential of extracellular matrix metalloproteinase inducer. Am J Pathol.

[51] Ke X, Fei F, Chen Y, Xu L, Zhang Z, Huang Q, Zhang H, Yang H, Chen Z, Xing J (2012). Hypoxia upregulates CD147 through a combined effect of HIF-1alpha and Sp1 to promote glycolysis and tumor progression in epithelial solid tumors. Carcinogenesis.

[52] Schwab LP, Peacock DL, Majumdar D, Ingels JF, Jensen LC, Smith KD, Cushing RC, Seagroves TN (2012). Hypoxia-inducible factor 1alpha promotes primary tumor growth and tumor-initiating cell activity in breast cancer. Breast Cancer Res.

[53] Louie E, Nik S, Chen JS, Schmidt M, Song B, Pacson C, Chen XF, Park S, Ju J, Chen EI (2010). Identification of a stem-like cell population by exposing metastatic breast cancer cell lines to repetitive cycles of hypoxia and reoxygenation. Breast Cancer Res.

[54] Conley SJ, Gheordunescu E, Kakarala P, Newman B, Korkaya H, Heath AN, Clouthier SG, Wicha MS (2012). Antiangiogenic agents increase breast cancer stem cells via the generation of tumor hypoxia. Proc Natl Acad Sci U S A.

[55] Oliveira-Costa JP, Zanetti JS, Silveira GG, Soave DF, Oliveira LR, Zorgetto VA, Soares FA, Zucoloto S, Ribeiro-Silva A (2011). Differential expression of HIF-1alpha in CD44 + CD24-/low breast ductal carcinomas. Diagn Pathol.

[56] Lock FE, McDonald PC, Lou Y, Serrano I, Chafe SC, Ostlund C, Aparicio S, Winum JY, Supuran CT, Dedhar S (2013). Targeting carbonic anhydrase IX depletes breast cancer stem cells within the hypoxic niche. Oncogene.

[57] Ricardo S, Vieira AF, Gerhard R, Leitao D, Pinto R, Cameselle-Teijeiro JF, Milanezi F, Schmitt F, Paredes J (2011). Breast cancer stem cell markers CD44, CD24 and ALDH1: expression distribution within intrinsic molecular subtype. J Clin Pathol.

[58] Diehn M, Cho RW, Lobo NA, Kalisky T, Dorie MJ, Kulp AN, Qian D, Lam JS, Ailles LE, Wong M, Joshua B, Kaplan MJ, Wapnir I, Dirbas FM, Somlo G, Garberoglio C, Paz B, Shen J, Lau SK, Quake SR, Brown JM, Weissman IL, Clarke MF (2009). Association of reactive oxygen species levels and radioresistance in cancer stem cells. Nature.

[CR59] The pre-publication history for this paper can be accessed here:http://www.biomedcentral.com/1471-2407/14/734/prepub

