# Supplementary material for: The basal epithelial marker P-cadherin associates with breast cancer cell populations harboring a glycolytic and acid-resistant phenotype
Source: BMC Cancer. 2014 Oct 1;14:734. doi: 10.1186/1471-2407-14-734 (PMC4190447; doi:10.1186/1471-2407-14-734)
Supplement: Supplementary file 1 — Additional file 1: Table S1: Clinical, pathological and immunohistochemical characteristics of the 473 primary invasive breast carcinomas*. Table S2. Association of P-cadherin, HIF-1α, GLUT1, CAIX, MCT1, MCT4 and CD147 and classic prognostic factors in breast cancer*. Table S3. Association of biomarkers and classical molecular markers used in breast cancer*. Table S4. Association between the hypoxia, glycolytic and acid-resistant phenotype markers within the series of invasive breast carcinomas*. Table S5. P-cadherin overexpression is associated with the expression of markers of hypoxia, glycolytic and acid-resistant phenotype in breast cancer*. (DOCX 80 KB) [file 12885_2014_4912_MOESM1_ESM.docx]

**Supplementary Table 1.** **Clinical, pathological and immunohistochemical characteristics of the 473 primary invasive breast carcinomas.***

| **Clinico-pathological and molecular characteristics** | **Age (years)** | Mean | 56.12 |  |
| --- | --- | --- | --- | --- |
|  |  | Min | 27 |  |
|  |  | Max | 89 |  |
|  |  | Missing | 2 |  |
|  | **Tumor size (mm)** | Mean | 31.6 |  |
|  |  | Min | 5 |  |
|  |  | Max | 150 |  |
|  |  | Missing | 243 |  |
|  |  |  | **Frequency (n)** | **Percentage (%)** |
|  | **Lymph-node metastasis** | Positive | 204 | 51 |
|  |  | Negative | 196 | 49 |
|  |  | Total | 400 | 100 |
|  |  | Missing | 73 | - |
|  | **Histological grade** | I | 155 | 33.2 |
|  |  | II | 200 | 42.8 |
|  |  | III | 112 | 24 |
|  |  | Total | 467 | 100 |
|  |  | Missing | 6 | - |
|  | **Molecular subtypes** | Luminal A | 262 | 57.3 |
|  |  | Luminal B | 14 | 3.1 |
|  |  | HER2 OE | 56 | 12.3 |
|  |  | Basal | 83 | 18.2 |
|  |  | Unclassified | 42 | 9.2 |
|  |  | Total | 457 | 100 |
|  |  | Missing | 16 | - |
|  | **ER** | Positive | 275 | 58.4 |
|  |  | Negative | 196 | 41.6 |
|  |  | Total | 471 | 100 |
|  |  | Missing | 2 | - |
|  | **PgR** | Positive | 177 | 37.9 |
|  |  | Negative | 290 | 62.1 |
|  |  | Total | 467 | 100 |
|  |  | Missing | 6 |  |
|  | **HER2** | Positive | 69 | 14.9 |
|  |  | Negative | 393 | 85.1 |
|  |  | Total | 462 | 100 |
|  |  | Missing | 11 | - |
|  | **Ki67** | >20 | 98 | 39.5 |
|  |  | <20 | 150 | 60.5 |
|  |  | Total | 248 | 100 |
|  |  | Missing | 225 | - |
| **Biomarkers** | **P-cadherin** | Positive | 145 | 31.0 |
|  |  | Negative | 323 | 69.0 |
|  |  | Total | 468 | 100 |
|  |  | Missing | 5 | - |
|  | **HIF-1α** | Positive | 104 | 33 |
|  |  | Negative | 211 | 67.0 |
|  |  | Total | 315 | 100 |
|  |  | Missing | 158 |  |
|  | **GLUT1** | Positive | 140 | 42.8 |
|  |  | Negative | 187 | 57.2 |
|  |  | Total | 327 | 100 |
|  |  | Missing | 146 | - |
|  | **CAIX** | Positive | 66 | 20.9 |
|  |  | Negative | 250 | 79.1 |
|  |  | Total | 316 | 100 |
|  |  | Missing | 157 | - |
|  | **MCT1** | Positive | 106 | 26 |
|  |  | Negative | 301 | 74 |
|  |  | Total | 407 | 100 |
|  |  | Missing | 66 | - |
|  | **MCT4** | Positive | 69 | 16.5 |
|  |  | Negative | 350 | 83.5 |
|  |  | Total | 419 | 100 |
|  |  | Missing | 54 | - |
|  | **CD147** | Positive | 24 | 11.1 |
|  |  | Negative | 193 | 88.9 |
|  |  | Total | 217 | 100 |
|  |  | Missing | 256 | - |

*****Characterization of the breast cancer series concerning age of the patients, tumor size, lymph-node metastasis, histological grade, molecular subtypes, ER, PgR, HER2 and Ki67 status, as well as expression of P-cadherin, HIF-1α, GLUT1, CAIX, MCT1, MCT4 and CD147.

|  | | **P-cadherin** | | | **HIF-1α** | | | **GLUT1** | | | **CAIX** | | | **MCT1** | | | **MCT4** | | | **CD147** | | |
| --- | --- | --- | --- | --- | --- | --- | --- | --- | --- | --- | --- | --- | --- | --- | --- | --- | --- | --- | --- | --- | --- | --- |
|  |  | **Positive** | **Negative** | **p** | **Positive** | **Negative** | **p** | **Positive** | **Negative** | **p** | **Positive** | **Negative** | **p** | **Positive** | **Negative** | **p** | **Positive** | **Negative** | **p** | **Positive** | **Negative** | **p** |
| **Histological Grade** | **I** | 21 (14.5%) | 133  (41.9%) | ***<0.0001*** | 30 (28.8%) | 91  (44.2%) | ***<0.0001*** | 35 (25.7%) | 84  (45.4%) | ***<0.0001*** | 13 (20.3%) | 100  (40.7%) | ***<0.0001*** | 29 (28.4%) | 99  (33.1%) | ***0.0011*** | 28  (40.6%) | 109 (31.7%) | *0.2663* | 0  (0%) | 44 (22.8%) | ***<0.0001*** |
|  | **II** | 57  (39.3%) | 140  (44.2%) |  | 39  (37.5%) | 88  (42.7%) |  | 53  (39%) | 76  (41.1%) |  | 23  (35.9%) | 101  (41%) |  | 35  (34.3%) | 142  (47.5%) |  | 24  (34.8%) | 153 (44.5%) |  | 6  (25%) | 96 (49.7%) |  |
|  | **III** | 67  (46.2%) | 44  (13.9%) |  | 35  (33.7%) | 27  (13.1%) |  | 48 (35.3%) | 25  (13.5%) |  | 28  (43.8%) | 45  (18.3%) |  | 38 (37.3%) | 58  (19.4%) |  | 17  (24.6%) | 82 (23.8%) |  | 18  (75%) | 53 (27.5%) |  |
| **Lymph-node metastasis** | **Positive** | 63  (47.7%) | 134  (50.2%) | *0.6438* | 44 (50.6%) | 90 (51.1%) | *0.9317* | 65 (53.7%) | 72  (45%) | *0.1477* | 32 (57.1%) | 102 (46.8%) | *0.1668* | 36 (39.6%) | 138 (53.5%) | ***0.0223*** | 31  (57.4%) | 147 (48.7%) | *0.2372* | 9 (37.5%) | 90 (52.9%) | *0.1566* |
|  | **Negative** | 69 (52.3%) | 133  (49.8%) |  | 43 (49.4%) | 86 (48.9%) |  | 56 (46.3%) | 88  (55%) |  | 24 (42.9%) | 116 (53.2%) |  | 55 (60.4%) | 120 (46.5%) |  | 23  (42.6%) | 155 (51.3%) |  | 15 (62.5%) | 80 (47.1%) |  |
| **Tumor Size**  **(mm)** | **Mean±SE**  **(n)** | 33.1**±**2.5 (81) | 30.8**±**1.7 (145) | *0.4375* | 34.8±3.2 (60) | 36.1±2.9 (67) | *0.764* | 30±2.7 (54) | 29.3±2.1 (67) | *0.8581* | 42.2±6.4 (21) | 27.2±1.4 (98) | ***0.0005*** | 35.5±3.6 (38) | 31.2±1.7 (165) | *0.2617* | 24.1±2.7  (15) | 32.5±1.6 (186) | *0.1454* | 29.4±3.5 (22) | 32.1±1.6 (180) | *0.5906* |

**Supplementary Table 2. Association of P-cadherin, HIF-1α, GLUT1, CAIX, MCT1, MCT4 and CD147 and classic prognostic factors in breast cancer***.

*Contingency table and chi-square test were applied to determine the statistical association between the biomarkers and the histological grade and lymph-node metastasis. Association between tumor size and the expression of these biomarkers were analyzed using ANOVA test considering a 95% confidence interval. *p* values less than *0.05* were considered statistically significant.

|  | | **P-cadherin** | | | **HIF-1α** | | | **GLUT1** | | | **CAIX** | | | **MCT1** | | | **MCT4** | | | **CD147** | | |
| --- | --- | --- | --- | --- | --- | --- | --- | --- | --- | --- | --- | --- | --- | --- | --- | --- | --- | --- | --- | --- | --- | --- |
|  |  | **Positive** | **Negative** | **p** | **Positive** | **Negative** | **p** | **Positive** | **Negative** | **p** | **Positive** | **Negative** | **p** | **Positive** | **Negative** | **p** | **Positive** | **Negative** | **p** | **Positive** | **Negative** | **p** |
| **Molecular Subtypes** | **Luminal A** | 35  (35.4%) | 223  (69.5%) | ***<0.0001*** | 49  (48.6%) | 122  (59.5%) | *0.076* | 73  (53.3%) | 121 (62.9%) | ***0.0001*** | 26  (40%) | 162  (66.7%) | ***<0.0001*** | 48  (46.2%) | 171 (58.5%) | ***<0.0001*** | 44  (65.6%) | 186 (54.4%) | ***0.032*** | 4  (18.2%) | 110  (58.8%) | ***<0.0001*** |
|  | **Luminal B** | 3  (3.0%) | 11  (3.4%) |  | 6  (5.9%) | 6  (2.9%) |  | 3  (2.2%) | 4  (2.2%) |  | 0  (0%) | 7  (2.9%) |  | 3  (2.9%) | 10  (3.4%) |  | 3  (4.5%) | 10  (2.9%) |  | 0  (0%) | 7  (3.7%) |  |
|  | **HER2 OE** | 38  (38.4%) | 18  (5.6%) |  | 16  (15.8%) | 27  (13.2%) |  | 12  (8.8%) | 20  (11%) |  | 10  (15.4%) | 20  (8.2%) |  | 8  (7.7%) | 44  (15.1%) |  | 3  (4.5%) | 52  (15.2%) |  | 4  (18.2%) | 28  (15%) |  |
|  | **Basal** | 23  (23.2%) | 27  (8.4%) |  | 23  (22.8%) | 27  (13.2%) |  | 41  (29.9%) | 17  (9.4%) |  | 24  (36.9%) | 33  (13.6%) |  | 38  (36.5%) | 37 (12.7%) |  | 15  (22.4%) | 59 (17.3%) |  | 12  (54.5%) | 29  (15.5%) |  |
|  | **Unclassified** | 0  (0%) | 42  (13.1%) |  | 7  (6.9%) | 23  (11.2%) |  | 8  (5.8%) | 19 (10.5%) |  | 5  (7.7%) | 21  (8.6%) |  | 7  (6.7%) | 30 (10.3%) |  | 2  (3%) | 35 (10.2%) |  | 2  (9.1%) | 13  (7%) |  |
| **ER** | **Positive** | 37  (25.9 %) | 233  (72.1%) | ***<0.0001*** | 56  (30.6%) | 46  (35.4%) | *0.3736* | 76  (54.7%) | 123 (65.8%) | ***0.0421*** | 25  (37.9%) | 168  (67.5%) | ***<0.0001*** | 50 (47.6%) | 182 (60.5%) | ***0.022*** | 48  (70.6%) | 194 (55.4%) | ***0.0205*** | 5  (20.8%) | 118 (61.5%) | ***0.0002*** |
|  | **Negative** | 106  (74.1%) | 90  (27.9%) |  | 127  (69.4%) | 84  (64.6%) |  | 63  (45.3%) | 64 (34.2%) |  | 41  (62.1%) | 81  (32.5%) |  | 55 (52.4%) | 119 (39.5%) |  | 20  (29.4%) | 156 (44.6%) |  | 19  (79.2%) | 74  (38.5%) |  |
| **PgR** | **Positive** | 24  (16.8%) | 150  (46.9%) | ***<0.0001*** | 34  (33.7%) | 82  (39%) | *0.3579* | 41  (29.3%) | 87 (46.5%) | ***0.016*** | 19  (28.8%) | 106  (42.4%) | ***0.0443*** | 30  (28.3%) | 121 (40.6%) | ***0.0245*** | 30  (43.5%) | 129  (36.5%) | *0.2402* | 2  (8.3%) | 78  (40.6%) | ***0.002*** |
|  | **Negative** | 119  (83.2%) | 170  (53.1%) |  | 67  (66.3%) | 128  (61%) |  | 99  (70.7%) | 100 (53.5%) |  | 47  (71.2%) | 144  (57.6%) |  | 76  (71.7%) | 177 (59.4%) |  | 39  (56.5%) | 224  (63.5%) |  | 22  (91.7%) | 114 (59.4%) |  |
| **HER2** | **Positive** | 41  (28.7%) | 28  (8.9%) | ***<0.0001*** | 22  (21.6%) | 32  (15.7%) | *0.2032* | 15  (10.9%) | 23 (12.5%) | *0.6705* | 10  (15.4%) | 26  (10.6%) | *0.2857* | 10  (9.7%) | 54 (18.4%) | ***0.0397*** | 6  (9%) | 61 (17.8%) | *0.0738* | 4  (17.4%) | 35  (18.4%) | *0.904* |
|  | **Negative** | 102  (71.3%) | 287  (91.1%) |  | 80  (78.4%) | 172  (84.3%) |  | 122 (89.1%) | 161 (87.5%) |  | 55  (84.6%) | 219  (89.4%) |  | 93  (90.3%) | 240 (81.6%) |  | 61  (91%) | 282 (82.2%) |  | 19  (82.6%) | 155 (81.6%) |  |
| **Ki67** | **High** | 43  (48.9%) | 51  (33.5%) | ***0.0141*** | 29  (43.3%) | 18  (24.7%) | ***0.0197*** | 33 (57.9%) | 26 (38.8%) | ***0.0339*** | 12  (54.5%) | 47  (53%) | *0.5214* | 22 (51.2%) | 59 (33.3%) | ***0.0297*** | 7  (8.9%) | 9 (6.5%) | *0.5258* | 14  (58.3%) | 65  (33.7%) | ***0.0179*** |
|  | **Low** | 45  (51.1%) | 101  (66.5%) |  | 38  (56.7%) | 55  (75.3%) |  | 24 (42.1%) | 41 (61.2%) |  | 10  (45.5%) | 53  (47%) |  | 21 (48.8%) | 118 (66.7%) |  | 72 (91.1%) | 129 (93.5%) |  | 10  (41.7%) | 128  (66.3%) |  |

**Supplementary Table 3. Association of biomarkers and classical molecular markers used in breast cancer*.**

*Association of P-cadherin, HIF-1α, GLUT1, CAIX, MCT1, MCT4 and CD147 expression with breast cancer molecular subtypes and the molecular biomarkers ER, PgR, HER2 and Ki67 in invasive breast carcinomas. Contingency table and chi-square test were applied to determine the statistical association of these parameters. *p* values less than *0.05* were considered statistically significant.

**Supplementary Table 4. Association between the hypoxia, glycolytic and acid-resistant phenotype markers within the series of invasive breast carcinomas***.

|  | | **HIF-1α** | | | **GLUT1** | | | **CAIX** | | | **MCT1** | | | **MCT4** | | |
| --- | --- | --- | --- | --- | --- | --- | --- | --- | --- | --- | --- | --- | --- | --- | --- | --- |
|  |  | **Positive** | **Negative** | **p** | **Positive** | **Negative** | **p** | **Positive** | **Negative** | **p** | **Positive** | **Negative** | **p** | **Positive** | **Negative** | **p** |
| **GLUT1** | **Positive** | 38  (69.1%) | 56  (34.6%) | ***<0.0001*** |  | | |  | | |  | | |  | | |
|  | **Negative** | 17  (30.9%) | 106  (65.4%) |  |  |  |  |  |  |  |  |  |  |  |  |  |
| **CAIX** | **Positive** | 24  (44.4%) | 26  (16.1%) | ***<0.0001*** | 52  (38.5%) | 14  (7,9%) | ***<0.0001*** |  | | |  | | |  | | |
|  | **Negative** | 30  (55.6%) | 135  (83.9%) |  | 83  (61,5%) | 163  (92,1%) |  |  |  |  |  |  |  |  |  |  |
| **MCT1** | **Positive** | 27  (27%) | 58  (28.7%) | *0.7554* | 52  (39.7%) | 26  (16.4%) | ***<0.0001*** | 26  (40.6%) | 52  (23.5%) | ***0.0069*** |  | | |  | | |
|  | **Negative** | 73  (73%) | 144  (71.3%) |  | 79  (60.3%) | 133  (83.6%) |  | 38  (59.4%) | 169  (76.5%) |  |  |  |  |  |  |  |
| **MCT4** | **Positive** | 26  (26.3%) | 30  (14.4%) | ***0.012*** | 38  (28.6%) | 26  (15%) | ***0.0039*** | 18  (27.7%) | 45  (19.4%) | *0.1486* | 29  (27.9%) | 38  (12.8%) | ***0.0004*** |  | | |
|  | **Negative** | 73  (73.7%) | 178  (85.6%) |  | 95  (71.4%) | 147  (85%) |  | 47  (72.3%) | 187  (80.6%) |  | 75  (72.1%) | 258  (87.2%) |  |  |  |  |
| **CD147** | **Positive** | 7  (11.1%) | 4  (5.6%) | *0.249* | 12  (23.5%) | 2  (3.6%) | ***0.0025*** | 8  (38.1%) | 7  (8.3%) | ***0.0005*** | 19  (45.2%) | 5  (2.9%) | ***<0.0001*** | 5  (31.3%) | 19  (9.6%) | ***0.0083*** |
|  | **Negative** | 56  (88.9%) | 67  (94.4%) |  | 39  (76.5%) | 53  (96.4%) |  | 13  (61.9%) | 77  (91.7%) |  | 23  (54.8%) | 167  (97.1%) |  | 11  (68.7%) | 179  (90.4%) |  |

*The association of the expression of the proteins of HIF-1α pathway (HIF-1α, GLUT1, CAIX, MCT1, MCT4 and CD147) was evaluated with contingency table and the chi-square test. *p* values less than *0.05* were considered statistically significant.

**Supplementary Table 5. P-cadherin overexpression is associated with the expression of markers of hypoxia, glycolytic and acid-resistant phenotype in breast cancer*.**

|  | | **HIF-1α** | | | **GLUT1** | | | **CAIX** | | | **MCT1** | | | **MCT4** | | | **CD147** | | |
| --- | --- | --- | --- | --- | --- | --- | --- | --- | --- | --- | --- | --- | --- | --- | --- | --- | --- | --- | --- |
|  |  | **Positive** | **Negative** | **p** | **Positive** | **Negative** | **p** | **Positive** | **Negative** | **p** | **Positive** | **Negative** | **p** | **Positive** | **Negative** | **p** | **Positive** | **Negative** | **p** |
| **P-cadherin** | **Positive** | 48  (46.2%) | 51  (24.4%) | ***<0.0001*** | 60  (43.5%) | 35  (18.8%) | ***<0.0001*** | 38  (57.6%) | 56  (22.7%) | ***<0.0001*** | 43  (41%) | 89  (29.7%) | ***0.0337*** | 24  (34.8%) | 109  (31.1%) | *0.5527* | 18  (75%) | 64  (33.3%) | ***<0.0001*** |
|  | **Negative** | 56  (53.8%) | 158  (75.6%) |  | 78  (56.5%) | 151  (81.2%) |  | 28  (42.4%) | 191  (77.3%) |  | 62  (59%) | 211  (70.3%) |  | 45  (65.2%) | 241  (68.9%) |  | 6  (25%) | 128  (66.7%) |  |

*Contingency table and the chi-square test were applied to determine the statistical association between the aberrant P-cadherin expression and the HIF-1α, GLUT1, CAIX, MCT1, MCT4 and CD147 expression, in a series of invasive breast carcinomas. *p* values less than *0.05* were considered statistically significant.
