# Supplementary material for: The basal epithelial marker P-cadherin associates with breast cancer cell populations harboring a glycolytic and acid-resistant phenotype
Source: BMC Cancer. 2014 Oct 1;14:734. doi: 10.1186/1471-2407-14-734 (PMC4190447; doi:10.1186/1471-2407-14-734)
Supplement: Supplementary file 2 — Additional file 2: Figure S1: P-cadherin is co-expressed with GLUT1 and CAIX in BT20 cell line. (PDF 179 KB) [file 12885_2014_4912_MOESM2_ESM.pdf]

Additional File 2

Supplemental Figure 1

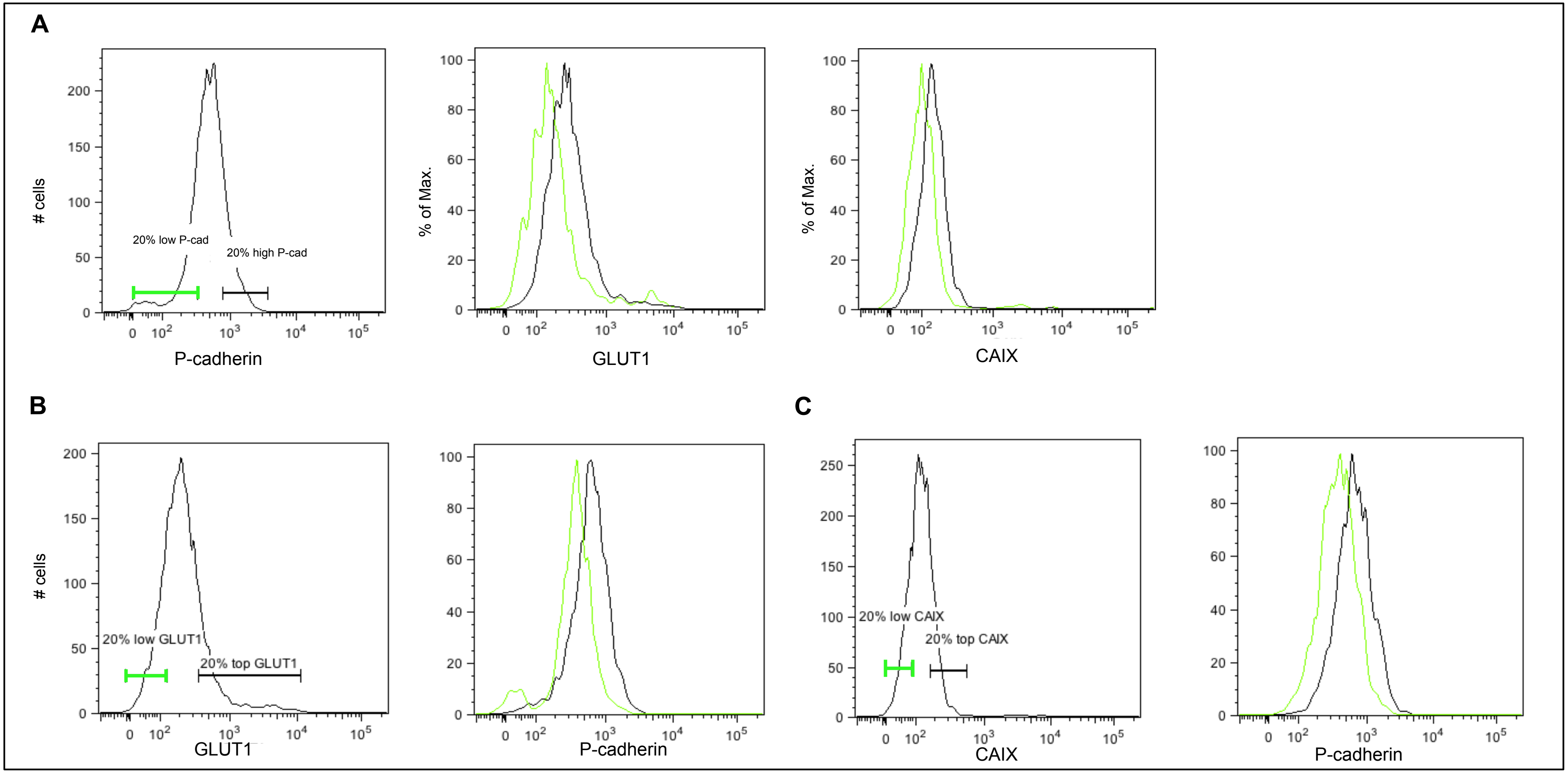

Supplementary Figure 1. P-cadherin is co-expressed with GLUT1 and CAIX in BT20 cell line. By flow cytometry analysis, we observe that the 20% of cells with the highest and lowest P-cadherin expression presents highest and lowest, respectively, expression of GLUT1 and CAIX (**A**). On the other hand, 20% of cells with the highest and lowest levels of GLUT1 and CAIX (**B** and **C**, respectively) expression also present highest and lowest P-cadherin expression.
